# Does T1-mapping in border-zone and/or remote regions can help to predict functional recovery after revascularization in chronic Coronary Total Occlusion (CTO) patients?

**DOI:** 10.1186/1532-429X-18-S1-O45

**Published:** 2016-01-27

**Authors:** Emile Youssof, Antoine Gerbay, Magalie Viallon, Alban Chazot, Karl Isaaz, Pierre Croisille

**Affiliations:** 1 Radiology, CHU Saint-Etienne, Université Jean Monnet, CREATIS CNRS 52220 INSERM 1040, Saint-Etienne, France; 2 Cardiology, CHU Saint-Etienne, Université J.Monnet, Saint-Etienne, France

## Background

Recanalization of chronic total occlusion (CTO-coronary occlusion > 3 months with a TIMI flow grade 0), is one of the most challenging PCI procedure with specific complications. Late-gadolinium enhancement imaging (LGE) is routinely used to assess viability by exploring transmural and circumferential extent of scar lesions in chronic ischemic regions, but is not able to further characterize adjacent and remote myocardium.

Border-zone (BZ) are noninfarcted normally perfused tissue regions adjacent to the infarct that are a major target of the always active remodeling process occuring after MI in chronic ischemic myocardium. Ultrastructural changes include myocyte elongation, myofiber rearrangement that trigger collagen deposition and may lead to depressed contractility. The aim of the study was therefore to evaluate the impact of myocardial fibrosis in BZ and remote regions using T1 and ECV mapping and to determine if their change affect prediction of functional recovery after revascularization of chronic total occlusion (CTO).

## Methods

58 patients were prospectively enrolled with CMR baseline. 37 patients were revascularized and all underwent CMR at 6 months with a follow-up coronary angiography within 5 ± 3 days. All CMR (1.5T Aera SIEMENS) examination included rest cine study, native (native-T1) and 15 min post- Gd injection (Gd-T1) MOLLI and 3D IR-TFL (LGE) sequences. Coronary CTO were performed using antegrade or retrograde techniques with drug eluting stent in all procedures. LV function and volumes were quantified (CMR42, Circle) as well as infarct size using a semi-quantitative grading scale. Native T1, post-Gd injection 15 min T1 and ECV maps were obtained with MOCO and automated coregistration. Myocardial segmentation of BZ regions, and remote regions was performed using CMRSegTools OsiriX plugin (Creatis, Lyon). Logistic regression modeling was used to compare the incremental value to infarct size (LGE) of: native-T1, Gd-T1, ECV mapping in BZ only, or BZ+remote regions in the prediction of contractile function recovery after revascularization.

## Results

After revascularization, ESV decreased from 40,5 ml/m^2^ (+/-16,7) to 36,2 ml/m^2^ (+/-13,8) (p = 0.06). EDV remained unchanged from 86,34 ml/m^2^ (+/-17,8) to 84,0 ml/m^2^ (+/-16,2) (p = 0,39). Revascularized patients had a significant LVEF improvement (p = 0.04). Compared to LGE only (Az = 0.68), estimate of ECV in BZ improved prediction to Az = 0.74 (p = 0.06), but the best model was obtained when estimate of ECV was considering BZ and remote regions Az = 078 (p < 0.01). Native T1 and Gd-T1 mapping alone did not provide comparable information in corresponding regions.

## Conclusions

In a CTO population, contractile functional recovery not solely depends on infarct size transmurality but is also related to the status of surrounding regions (BZ and remote regions) where ECV increase is likely to reflect interstitial fibrosis in non ischemic but remodeled regions.

**Figure 1:**
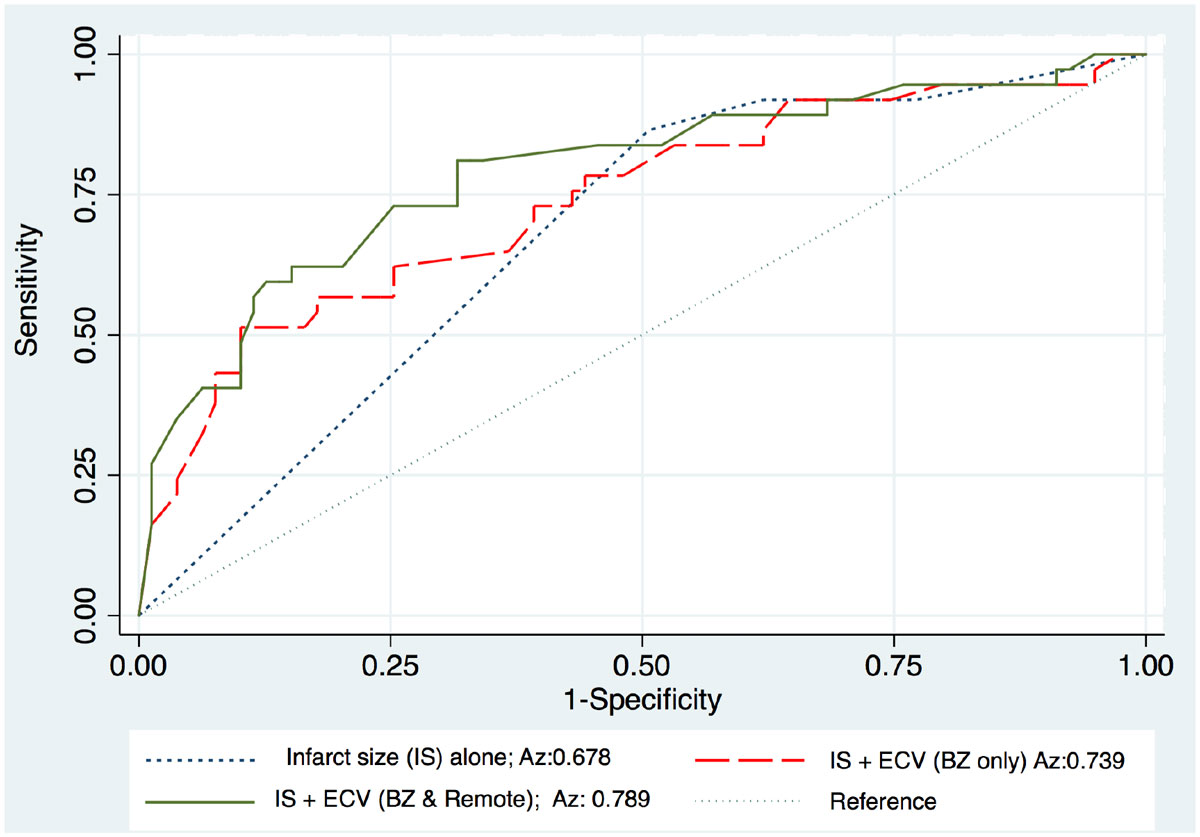
**Comparison of the logistic regression models and corresponding ROC curves for prediction of functional recovery after revascularization (Az = area under the curve)**.

